# CMR T1 reactivity in healthy adult hearts: a systematic review and meta-analysis

**DOI:** 10.3389/fcvm.2025.1627908

**Published:** 2025-11-13

**Authors:** Bingquan Wang, Hongyu Gu, LingLing Kong, Song Luo, Xiaoyi Deng

**Affiliations:** 1 Department of Radiology, Aoyang Hospital Affiliated to Jiangsu University, Zhangjiagang, Jiangsu, China; 2 Department of Radiology, Geriatric Hospital of Nanjing Medical University, Nanjing, Jiangsu, China

**Keywords:** T1 reactivity, T1 mapping, CMR, meta-analysis, healthy adult

## Abstract

**Background:**

Cardiac stress T1-mapping is an advanced magnetic resonance imaging technique that enables the detection of myocardial ischemia and coronary microvascular dysfunction without the need for gadolinium-based contrast agents (GBCAs). This review seeks to synthesize reported mean ΔT1 values from studies involving healthy adults, establish an approximate range of myocardial T1 reactivity in this population, and explore factors underlying the heterogeneity observed across different studies.

**Methods:**

PubMed, Web of Science, and Cochrane Central were searched for studies reporting myocardial T1 reactivity in healthy adult participants. The search strategy included terms such as: “Stress T1 ‘AND’ Mapping” OR “Stress T1 ‘AND’ Cardiovascular magnetic resonance” OR “Stress T1 ‘AND’ CMR” OR “T1 reactivity ‘AND’ Cardiovascular magnetic resonance” OR “T1 reactivity ‘AND’ CMR.” Use the Joanna Briggs Institute (JBI) quantitative critical appraisal tools for documentation quality assessment. The average value is summarized using the random effect model, and heterogeneity was assessed by using the inconsistency factor (*I*^2^). A sensitivity analysis of the incorporated research was carried out using a one-by-one exclusion method. Subgroup analysis and regression analysis were used to determine the causes of heterogeneity.

**Results:**

This systematic review of T1 reactivity included 10 articles (11 study groups), with 226 participants (mean age, 52.21 years; 56.19% men [127 of 226]). The pooled mean of ΔT1 was 6.22% (95% confidence interval [CI]: 5.60, 6.84). *I*^2^ was 89.07%. The mean ΔT1 was 5.42% (95% CI: 4.77, 6.07) at modified Look Locker Inversion recovery (MOLLI) and 6.82% (95% CI: 5.98, 7.66) at shortened modified Look Locker inversion recovery (SHMOLLI). There was substantial heterogeneity in both pools (*I*^2^ = 80.16% and *I*^2^ = 83.89% at MOLLI and SHMOLLI, respectively). There is a statistically significant statistical difference in ΔT1 between MOLLI and SHMOLLI (*p* = 0.01). Pooled meta-regression analyses of all health study cohorts revealed age as an significantly associated with ΔT1 value variations (*p* = 0.038).

**Conclusions:**

This analysis summarizes the pooled means and CI of T1 reactivity in healthy adult participants. Significant heterogeneity was observed, highlighting the need to standardize cardiac MRI protocols and to investigate factors influencing T1 reactivity.

**Systematic Review Registration:**

identifier CRD42024568804.

## Introduction

Stress CMR is the most sensitive non-invasive imaging technique for diagnosing ischemic cardiomyopathy and can provide essential diagnostic and prognostic information (1). T1 mapping, a non-contrast parametric mapping technique in CMR, allows quantitative tissue characterization at the pixel level. While late gadolinium enhancement (LGE) is effective in detecting focal myocardial fibrosis, its utility is limited in assessing diffuse interstitial fibrosis. In contrast, T1 mapping techniques—such as native T1 values and extracellular volume (ECV) fraction—enable the identification of myocardial edema and diffuse fibrosis (2–4). The combination of cardiac stress testing with T1 mapping allows accurate detection of myocardial ischemia and coronary microvascular dysfunction without the administration of GBCAs. Previous studies have shown that adenosine-stress T1-mapping CMR effectively discriminates between normal, ischemic, infarcted, and remote myocardial tissues without the need for GBCAs (2, 5, 6).

During stress, coronary vasodilation leads to an increase in myocardial blood volume (MBV) within the myocardium, which can be observed using T1-mapping by assessing the partial volume effect of blood T1 (7). This phenomenon is referred to as T1 reactivity (ΔT1), where ΔT1 = (stress T1—native T1)/native T1 (8). It should be noted that ΔT1 does not represent a direct quantitative measurement. T1 reactivity is a reliable parameter for assessing myocardial blood volume change on CMR, comparable to semiquantitative myocardial perfusion reserve index (MPRI) or quantitative myocardial perfusion reserve (MPR) methods (9, 10). However, contemporary studies reveal that T1 signals reflect a more nuanced physiological phenomenon than can be explained solely by myocardial blood volume. When interpreting these signals, it is essential to account for other potential contributing factors—particularly stress-induced, reversible interstitial edema and alterations in membrane permeability (2, 11). Therefore, the observed changes in T1 values should be regarded as a comprehensive measure of the “holistic stress response of the myocardial tissue environment,” rather than merely a pure surrogate for blood flow.

Defining the normal range of healthy myocardium is a prerequisite for distinguishing abnormal myocardium. Therefore, the current priority lies in systematically elucidating and quantifying the range of myocardial ΔT1 values along with their influencing factors, thereby establishing the clinical utility of ΔT1 as a reliable quantitative biomarker for both routine cardiac evaluation and advanced scientific investigations. The aims of this review are to summarize reported mean ΔT1 values from studies of healthy adults, to establish an approximate range of myocardial T1 reactivity, and to explain the sources of heterogeneity.

## Materials and methods

This meta-analysis followed the PRISMA (an updated guideline for reporting systematic reviews) statement (12). The review protocol is registered with PROSPERO (CRD42024568804).

### Search strategy

Two independent reviewers (B.W., a cardiology fellow with 8 years of experience, and H.G., a senior doctor member with over 15 years of experience) systematically searched PubMed, Web of Science, and Cochrane Central for articles of myocardial ΔT1 in healthy subjects. The search terms used were “stress AND T1 mapping” OR “stress T1 AND cardiac magnetic resonance” OR “stress T1 AND CMR” OR “stress reactivity.” Instead of using the search terms together, they were used individually. Date limits were not set. The search was performed on January 1, 2025. Reviewers reviewed reference lists of eligible articles to identify additional articles not found in database searches.

### Eligibility criteria

Two reviewers (B.W. and H.G.) assessed the titles and abstracts of possibly relevant research for suitability before retrieving the full article. Any disagreements over the article's eligibility are resolved by conversation with a third reviewer (S.L.). Studies were included if they reported myocardial T1 reactivity in healthy subjects using CMR. Every study that included a group of at least ten healthy adults over the age of 18—with no overt heart disease symptoms, no known heart disease, and negative CMR imaging findings—was considered for inclusion in the analysis. Research involving individuals who were thought to have a low risk of heart disease or that had heart disease risk factors, such as hypertension, hyperlipidemia, diabetes mellitus, or tobacco use, was also included. Excluded from consideration were studies involving athletes or those who had CMR following exercise stress. Exclusion criteria included reviews, editorials, abstracts, conference presentations, research on animals, studies not directly related to the issue of interest, and studies published in languages other than English.

### Data collection

Two reviewers (B.W. and S.L.) carried out the data abstraction and study review. Because the clinical measurements of interest were T1 reactivity in healthy participants, authors directly abstracted myocardium native T1, myocardium stress T1, and myocardium ΔT1 from the text and tables of entirely reviewed articles. Details on study size and demographics were extracted from the text and tables. Comprehensive extraction of details of the CMR program used, including vendor, field strength, pulse sequence scheme, flip angle, repetition time, echo time, stress agent, and stress time. Every data collection was viewed as a distinct research group if an article provided data for different field strengths.

### Quality assessment

Using the Joanna Briggs Institute (JBI) quantitative critical appraisal tool (checklist for analytical cross-sectional studies) (13), two authors (B.W. and H.G.) independently evaluated the quality of the included studies. In the event of a disagreement, a third author (S.L.) was involved. The checklist consisted of eight items, as follows: (1) Were the criteria for inclusion in the sample clearly defined? (2) Were the study subjects and the setting described in detail? (3) Was the exposure measured in a valid and reliable way? (4) Were objective, standard criteria used for the measurement of the condition? (5) Were confounding factors identified? (6) Were strategies to deal with confounding factors stated? (7) Were the outcomes measured in a valid and reliable way? (8) Was appropriate statistical analysis used? Total scores ranged from 0 to 8, and the responses were scored 0 for “no” (×) and 1 for “yes” (√). The studies were classified as low quality with a high risk of bias if the overall score was ≤4.

### Data analysis

The summary means and CIs of native T1, stress T1 and ΔT1 were calculated by using the DerSimonian-Laird random-effect model (14) weighted by the inverse of the variance. For every meta-analysis, the *I*^2^ statistic was provided, and outcomes were considered heterogeneous if the value was more than 50%. Subgroup analyses and meta-regression analyses were employed to identify variables that significantly influenced native T1, stress T1, and ΔT1 results, thereby explaining the observed heterogeneity.” Sensitivity analysis using one-by-one removal methods. Observing how modifications to the combined findings affect the main meta-analysis's result to evaluate the summarized results' robustness. Small study and publishing biases were investigated using funnel plots and the Egger test. Stata MP 18.0 software for all meta-analyses. Statistical significance was defined as two-sided *p* values <0.05.

## Results

### Results of the literature search

**Figure 1:**
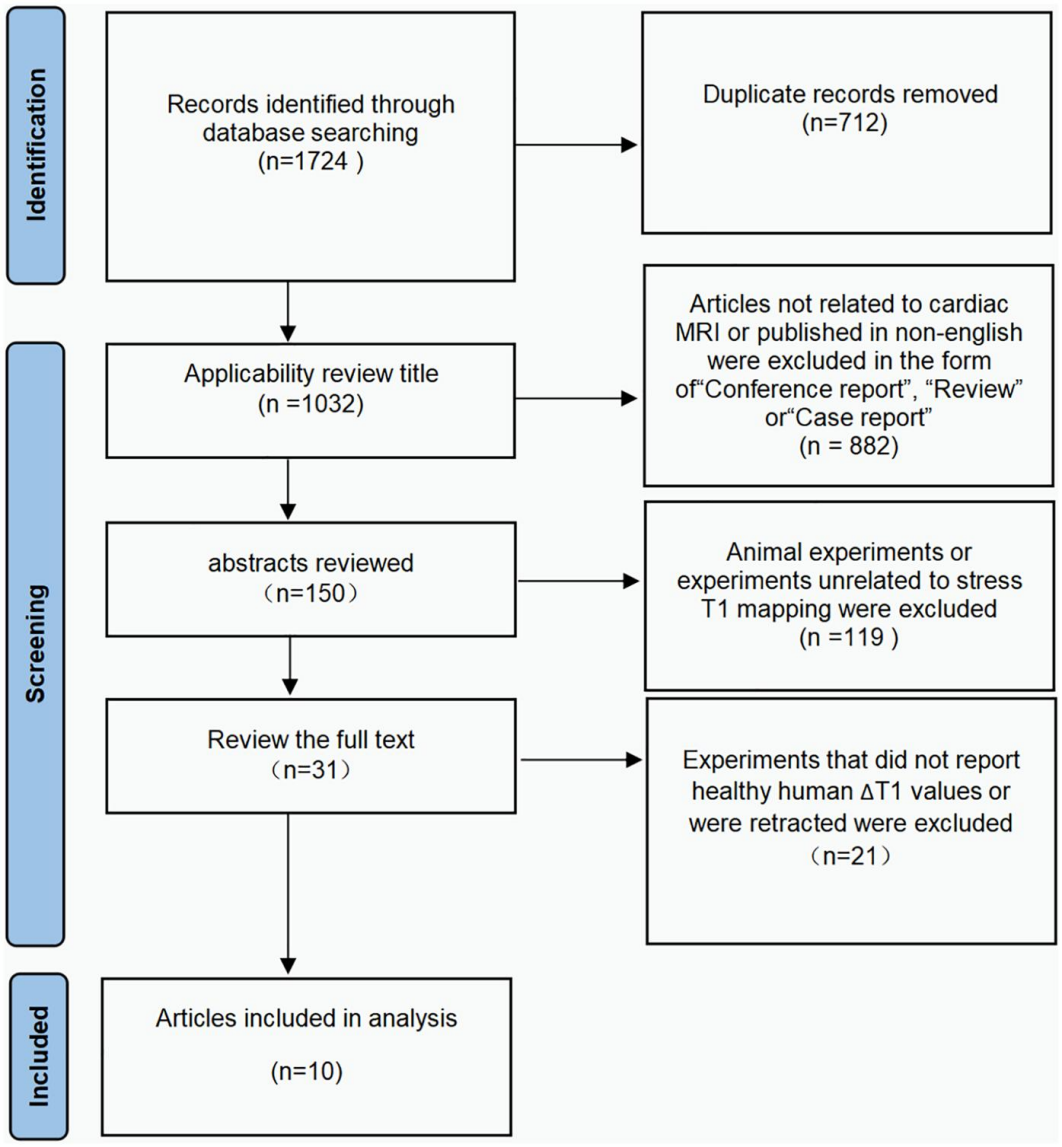
Preferred reporting items for systematic reviews and meta-analyses (PRISMA) flow diagram of study selection.

Study characteristics are shown in Table 1. The database search identified 1,724 articles, 617 from PubMed, 957 from Web of Science, and 151 from Cochrane Central. Removal of 712 duplicate articles. The titles of the 1,032 unique articles were reviewed for relevance, and 150 met the criteria for abstract review. After reviewing the abstracts, 31 publications satisfied the criteria for full-text review. Ultimately, a total of 10 articles matched this meta-analysis. Our search considered all vendors and pulse sequence, but the number of studies needed for pooled analysis was limited to studies utilizing Siemens (Erlangen, Germany) scanners (one study that used Philips as the sole vendor was excluded to avoid potential bias in the statistical analysis, as its inclusion could undermine the validity of the results) and merely modified Look Locker Inversion recovery (MOLLI) and shortened modified Look Locker inversion recovery (SHMOLLI) procedures. Details of the search strategy are presented in Figure 1.

**Table 1:** Characteristics and technical data of the included studies.

Study	Year	Study type	Country	Technique	Field strength (T)	Sequence	Flip angle (°)	TR (ms)	TE (ms)	Study design
Burrage, 1	2021	Single center	UK	Siemens	1.5	ShMOLLI	35	1.96	0.98	Prospective
Burrage, 2	2021	Single center	UK	Siemens	1.5	ShMOLLI	35	1.96	0.98	Prospective
Dirkjan, 1	2017	Single center	Netherlands	Siemens	1.5	MOLLI	35	-	-	Prospective
Dirkjan, 2	2016	Single center	Netherlands	Siemens	1.5	MOLLI	35	-	-	Prospective
Hisanori	2023	Single center	Japan	Siemens	3.0	MOLLI	35	349	1.1	Retrospective
Levelt	2017	Single center	UK	Siemens	3.0	ShMOLLI	35	1.96	0.98	Prospective
Li	2024	Single center	China	Siemens	3.0	MOLLI	35	330	0.98	Prospective
Liu; 1.5T	2016	Single center	UK	Siemens	1.5	ShMOLLI	35	1.96	0.98	Prospective
Liu; 3.0T	2016	Single center	UK	Siemens	3.0	ShMOLLI	35	1.96	0.98	Prospective
Mahmod	2014	Single center	UK	Siemens	3.0	ShMOLLI	35	1.96	0.98	Prospective
Sree	2020	Multi-center	Austral	Siemens	3.0	ShMOLLI	35	1.96	0.98	Prospective

Study	Year	No. of healthy adults in study	Age (years)	Male (%)	Stress agent	Stress time	Resting heart rate (bpm)	BMI (kg/m^2^)	Native T1 (ms)	Stress T1 (ms)	ΔT1 (%)
Burrage, 1	2021	10	32 ± 5.9	50%	Regadenoson	30 s	-	-	931 ± 22	1,008 ± 24	8.2 ± 0.8
Burrage, 2	2021	10	35 ± 8	50%	Regadenoson	15–20 s	-	-	921 ± 19	979 ± 28	6.4 ± 1.7
Dirkjan, 1	2017	24	65 ± 11	46%	Regadenoson	3 min	82 ± 18	-	971 ± 33	1,023 ± 43	5.4 ± 2.4
Dirkjan, 2	2016	50	66 ± 11	44%	Adenosine	3 min	76 ± 15	-	977 ± 40	1,018 ± 40	4.3 ± 2.8
Hisanori	2023	14	58.6 ± 4.3	71.4%	ATP	3 min	70 ± 3	22.2 ± 0.55	1,197 ± 10	1,248 ± 10	5.6 ± 0.5
Levelt	2017	16	51 ± 9	53%	Adenosine	3 min	58 ± 10	25.8 ± 4.2	1,194 ± 26	1,273 ± 44	6.6 ± 2.6
Li	2024	55	63 ± 5.97	47.3%	Adenosine	4 min	71.44 ± 3.76	23.74 ± 3.49	1,212.83 ± 14.32	1,212.83 ± 18.89	6.17 ± 1.9
Liu;1.5T	2016	10	33 ± 10	70%	Adenosine	3–6 min	68 ± 12	25.8 ± 4.2	954 ± 19	1,013 ± 23	6.2 ± 0.5
Liu;3.0T	2016	10	36 ± 11	70%	Adenosine	3–6 min	64 ± 15	25 ± 3	1,189 ± 34	1,264 ± 28	6.3 ± 1.1
Mahmod	2014	16	63.3 ± 3.4	50%	Adenosine	3 min	64 ± 11	27.0 ± 3.8	1,168 ± 27	1,238 ± 54	6.0 ± 4.2
Sree	2020	11	64 ± 7	63.6%	Adenosine	120 s	-	-	1,153 ± 33	1,245 ± 38	8.0 ± 2.9

Values are expressed as mean ± standard deviation or as percentage of subjects. UK, United Kingdom; MOLLI, modified look-locker inversion recovery; ShMOLLI, shortened MOLLI; BMI, body mass index; ATP, adenosine triphosphate.

### Study characteristics

Study characteristics are shown in Table 1. The 10 articles on T1 reactivity included 11 study groups (5 study groups at 1.5T, 6 study groups at 3.0T) and 226 participants. Mean age, 52.52 years (95% CI: 43.92, 60.50), 56.19% men (127 of 226). The largest study, conducted by Li et al. (2), included 55 participants.

### Pooled analysis of native T1 and stress T1

**Figure 2:**
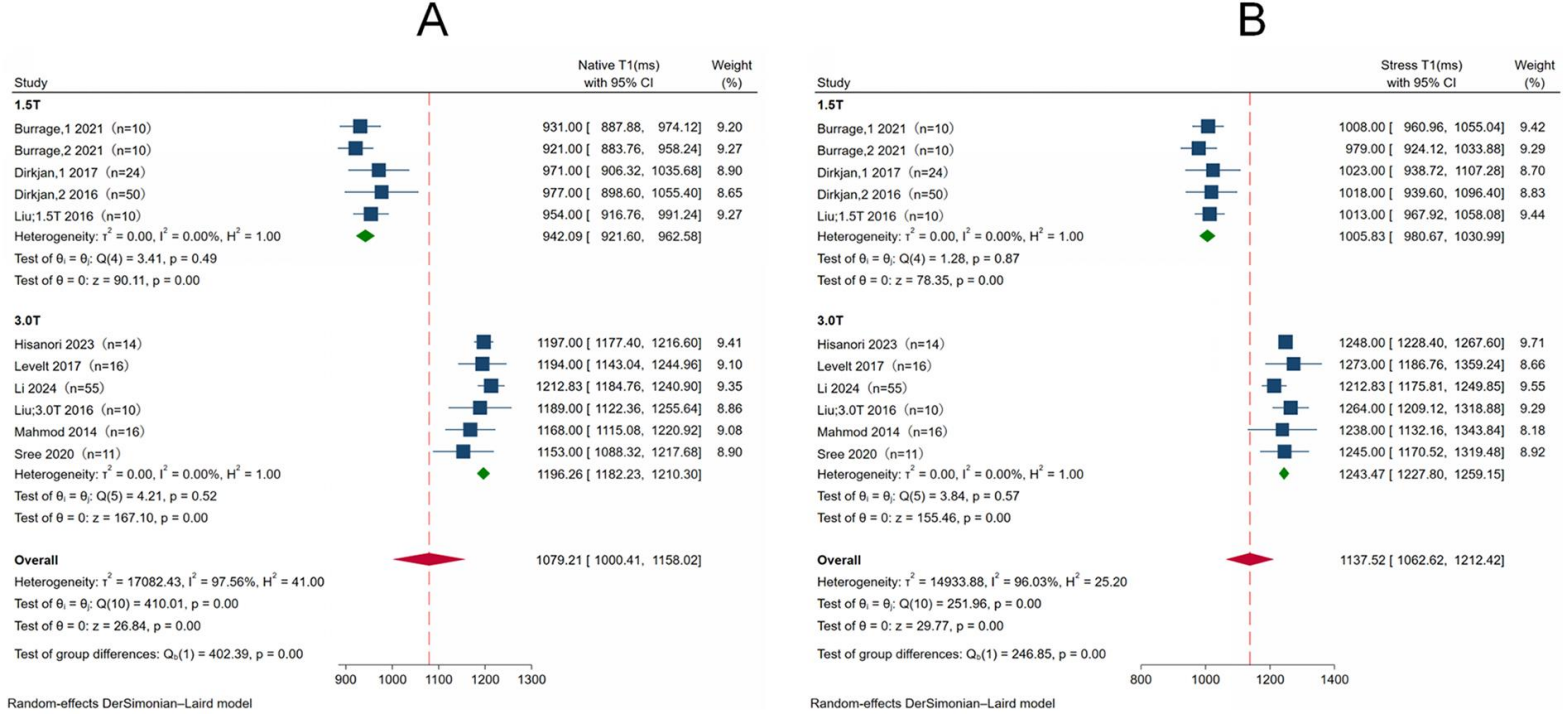
Forest plot of native T1 **(A)** and stress T1 **(B)** subgroup analysis between 1.5T and 3.0T. The meta-analysis showed a significant difference between 1.5T and 3.0T in terms of Native T1 (1,079.21, 95 ms, CI 1,000.41–1,158.02, *p* < 0.001) and Stress T1 (1,137.52 ms, 95% CI 1,062.62–1,212.42, *p* < 0.001). CI, confidence interval.

The mean native T1 was 942.09 ms (95% CI: 921.60, 962.58) at 1.5T and 1,196.26 ms (95% CI: 1,182.23, 1,210.30) at 3.0T. There was no significant heterogeneity in both pools. *I*^2^ was 0% in both the 1.5-T and 3.0-T pools. Native T1 values were longer at 3.0T than at 1.5T (*p* < 0.001). Figure 2A shows significant differences in the pooled mean native T1 by field strength. The summary mean of all stress T1 study groups studied was 1,137.52 ms (95% CI: 1,062.62, 1,212.42), *I*^2^ was 96.03%. There was no significant heterogeneity in both pools. *I*^2^ was 0% in both the 1.5-T and 3.0-T pools. Stress T1 values were longer at 3.0T than at 1.5T (*p* < 0.001). Figure 2B shows significant differences in the pooled mean stress T1 by field strength.

### Pooled analysis of ΔT1

**Figure 3:**
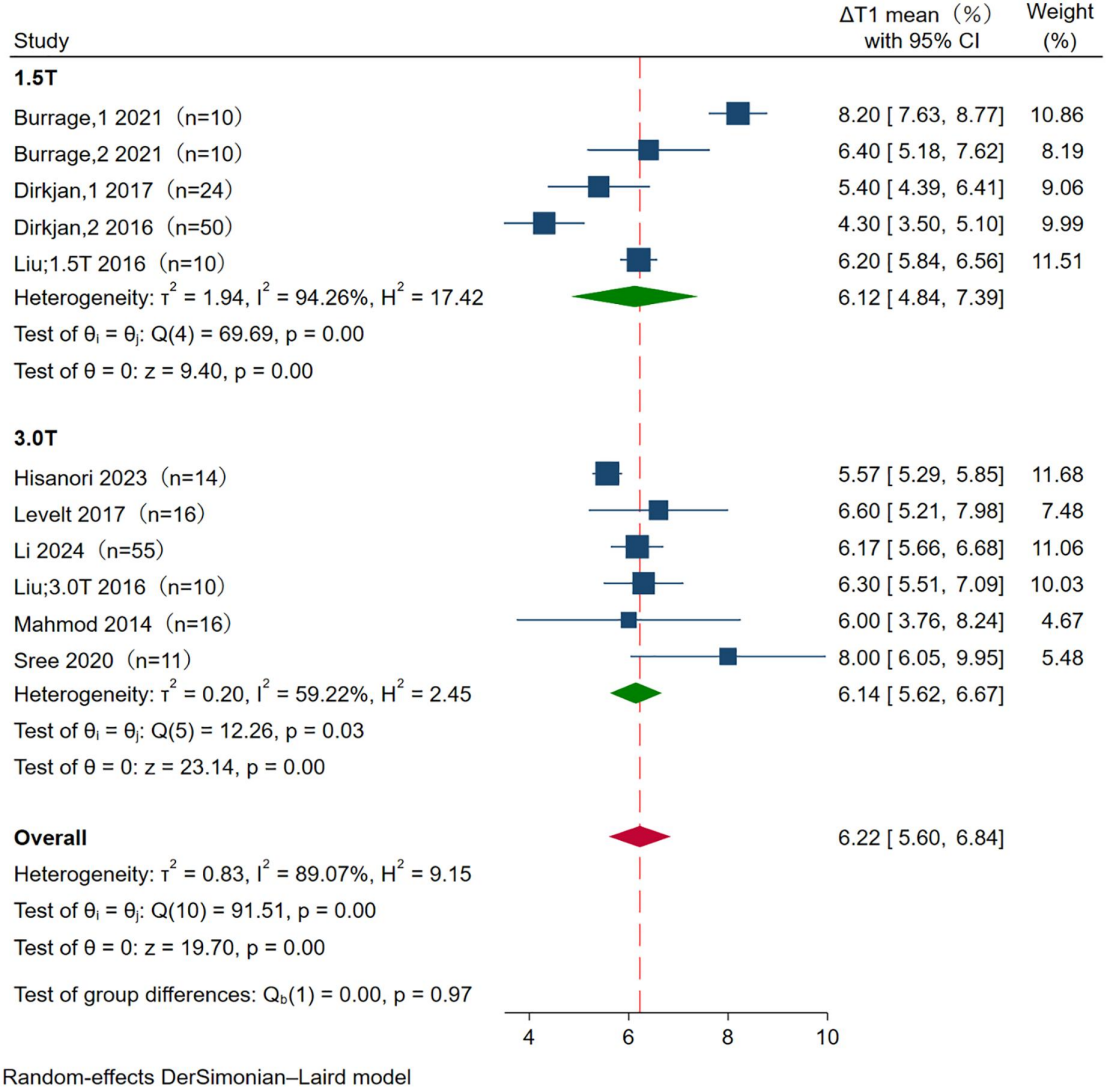
Forest plot of subgroup analysis for ΔT1 between 1.5T and 3.0T. The meta-analysis showed no significant difference between 1.5T (6.12%, 95% CI 4.84–7.39) and 3.0T (6.14%, 95% CI 5.62–6.67) in ΔT1, with *p* = 0.97. CI, confidence interval.

The summary mean of all ΔT1 study groups studied was 6.22% (95% CI: 5.60, 6.84), *I*^2^ was 89.07%. The mean ΔT1 was 6.12% (95% CI: 4.84, 7.39) at 1.5T, and 6.14% (95% CI: 5.62, 6.67) at 3.0T. There was substantial heterogeneity in both pools (*I*^2^ = 94.26% and *I*^2^ = 59.22% at 1.5-T and 3-T, respectively). There is no significant statistical difference in ΔT1 between 1.5T and 3.0T (*p* = 0.97) (Figure 3).

**Figure 4:**
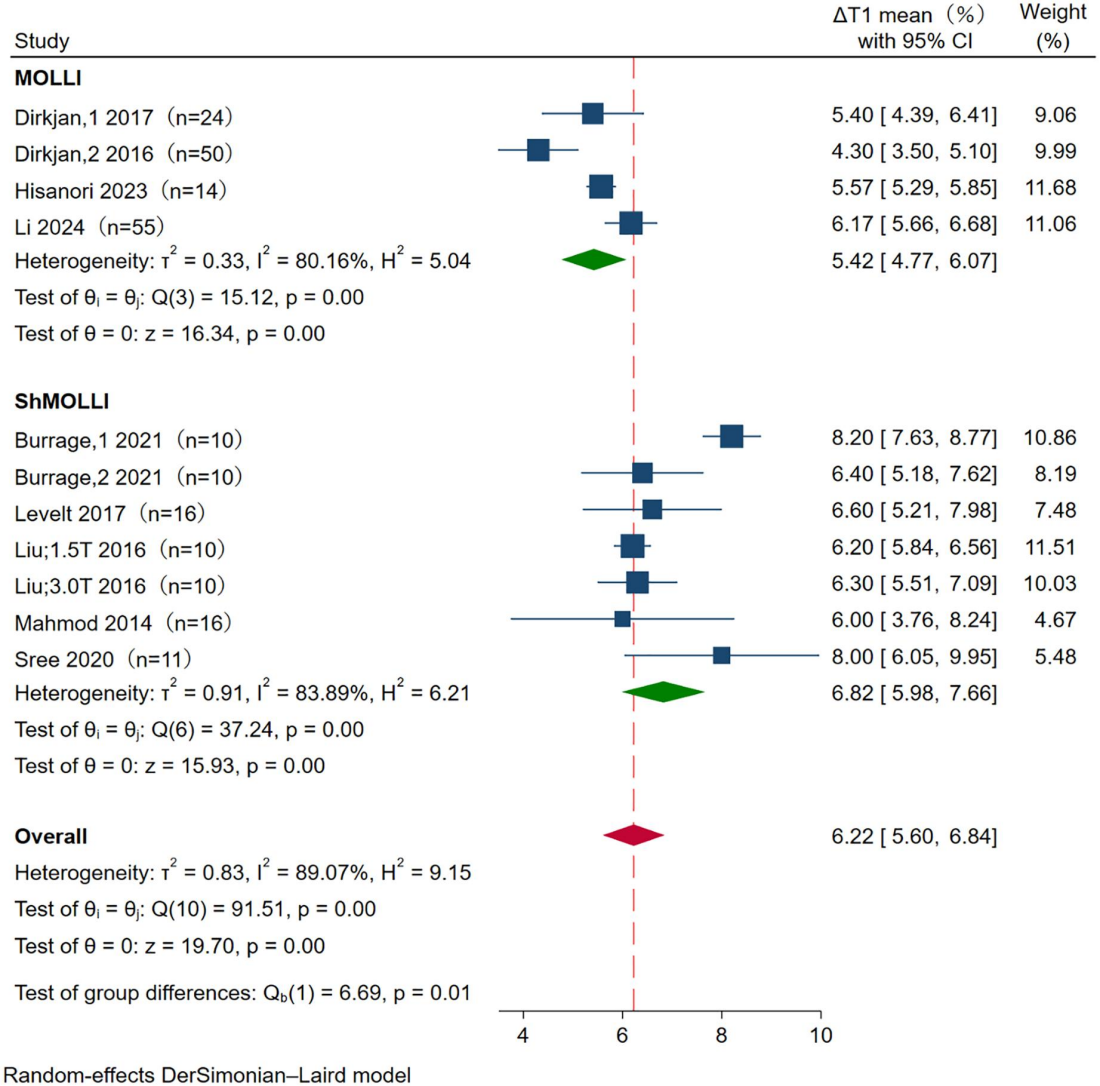
Forest plot of subgroup analysis for ΔT1 between MOLLI and ShMOLLI. The meta-analysis showed a significant difference between MOLLI (5.42%, 95% CI 4.77–6.07) and ShMOLLI (6.82%, 95% CI 5.98–7.66) in ΔT1, with *p* < 0.001. CI, confidence interval.

The mean ΔT1 was 5.42% (95% CI: 4.77, 6.07) at MOLLI, and 6.82% (95% CI: 5.98, 7.66) at SHMOLLI. There was substantial heterogeneity in both pools (*I*^2^ = 80.16% and *I*^2^ = 83.89% at MOLLI and SHMOLLI, respectively). There is a statistically significant statistical difference in ΔT1 between MOLLI and SHMOLLI (*p* = 0.01) (Figure 4).

In our study on the application of the ShMOLLI sequence, we performed a subgroup analysis of various load medications. The summary mean of ΔT1 was 7.38 (95% CI: 5.63, 9.14) in regadenoson and 6.28 (95% CI: 5.97, 6.59) in adenosine (*I*^2^ = 85.49% and *I*^2^ = 0%, respectively). There is no significant statistical difference between the two study groups (*p* = 0.22) (Supplementary Figure S1).

Pooled meta-regression analyses of all health study cohorts revealed age to be significantly associated with ΔT1 value variations (*p* = 0.038), whereas the models demonstrated no statistically significant association between ΔT1 changes and cohort gender composition (*p* = 0.662).

### Sensitivity analyses

A one-by-one exclusion approach was employed to do a sensitivity analysis on the included study. The pool value ranges from 5.92 to 6.43, which is within the original range (5.60–6.84) (Supplementary Figure S2). The study's findings demonstrate strong robustness.

### Publication bias

**Figure 5:**
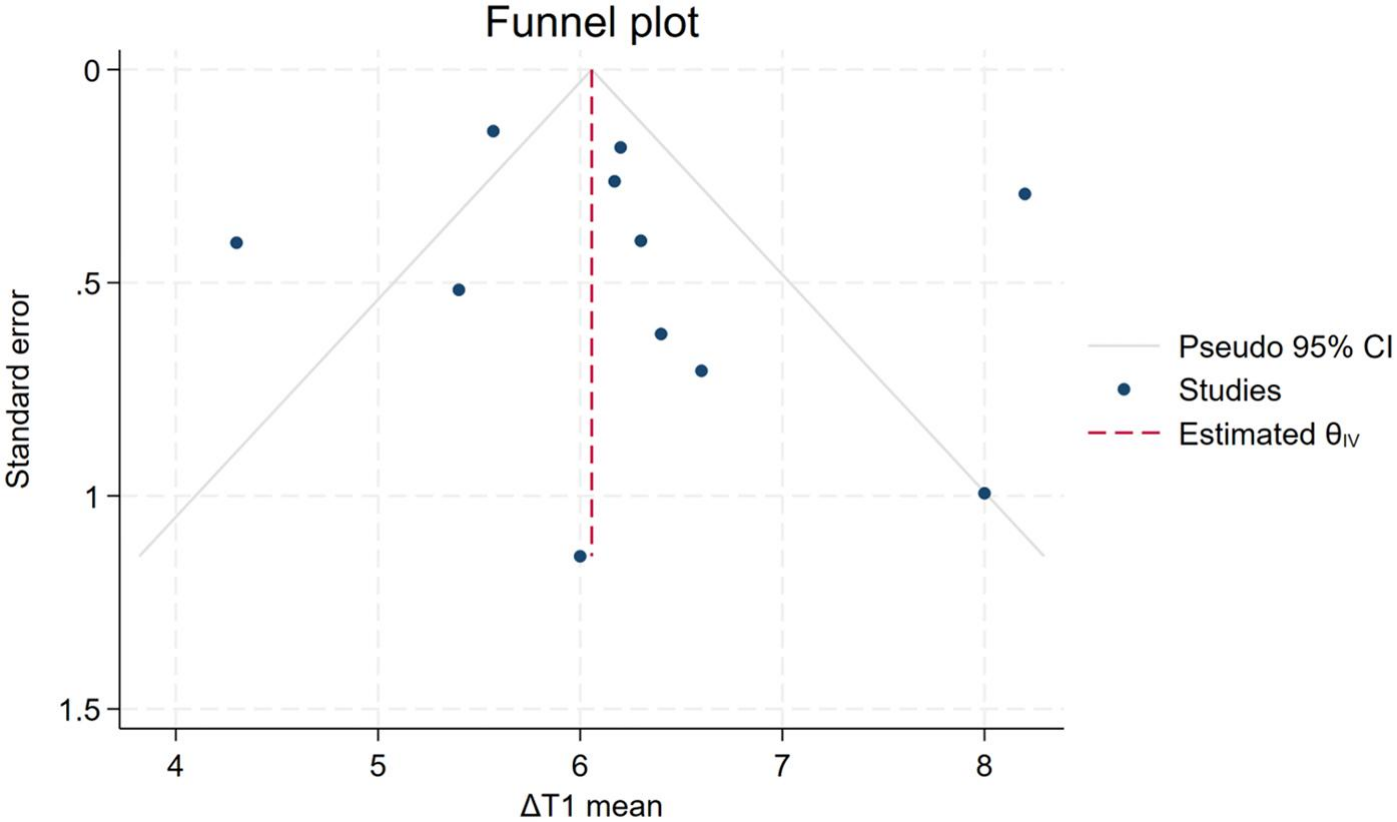
Funnel chart of ΔT1 for healthy adults. Each dot represents a study; the *y*-axis represents study precision [standard error (S.E.) of effect size] and the *x*-axis the effect size. Large studies appear toward the top of the graph and tend to cluster near the mean effect size. Small studies appear toward the bottom of the graph and are dispersed across a range of values since there is more sampling variation in effect size estimates. The outer dashed lines indicate the triangular region within which 95% of studies are expected to lie in the absence of biases and heterogeneity.

The funnel plot demonstrates a visually symmetrical distribution of study points along the central axis, with individual studies evenly dispersed on both sides (Figure 5). Egger's test (*Z* = 0.58, *p* = 0.56) indicated the absence of significant publication bias in this meta-analysis.

### Quality assessment

All of the studies that were used with the JBI critical assessment method are included in Table 2. No study met all criteria for quality (7 score points, 1 article; 6 score points, 7 articles; 5 score points, 2 articles). The majority of research don't include a thorough explanation of the information (including demographics, location, and time period) collected from the healthy adults. In all studies, no recognized criteria were used to define healthy adults, demonstrating shortcomings in technique, statistical analysis, and sample size.

**Table 2:** Screening parameters, according to the prevalence checklist of related JBI critical appraisal tool and the resulting score for risk of bias of each.

Study	Q1	Q2	Q3	Q4	Q5	Q6	Q7	Q8	Total score
Burrage, 1 2021	×	一	×	√	√	√	×	√	5
Burrage, 2 2021	√	一	√	√	√	√	×	√	6
Dirkjan, 1 2017	√	×	√	√	√	√	×	√	6
Dirkjan, 2 2018	√	×	√	√	√	√	×	√	6
Hisanori, 2023	×	√	×	√	√	√	×	√	5
Levelt, 2017	√	×	√	√	√	√	×	√	7
Li, 2024	√	×	√	√	√	√	×	√	6
Liu, 2016	√	×	√	√	√	√	×	√	6
Mahmod, 2014	√	×	√	√	√	√	×	√	6
Sree, 2020	√	×	√	√	√	√	×	√	6

Total scores ranged from 0 to 8 and the responses were scored 0 for “No” (×), 0 for “Unclear” (一) and 1 for “Yes” (√). The studies were classified as lowquality, high-risk of bias, if the overall score was ≤4.

## Discussion

The pooled mean ΔT1 across all study groups was 6.22% (95% CI: 5.60, 6.84), with an *I*^2^ value of 89.07%. Substantial heterogeneity was observed in the pooled mean ΔT1 among healthy adults, which remained significant even after adjusting for multiple factors. Part of this heterogeneity was explained by pulse sequence and age.

Given that T1 mapping is known to be influenced by magnetic field strength, differences in native and stress T1 values between 1.5T and 3.0T were expected (*p* < 0.001 vs. *p* < 0.001) (15). However, the effect of magnetic field strength on T1 responsiveness remains unclear. Subgroup analysis revealed no statistically significant difference in pooled ΔT1 values between the 1.5T and 3.0T groups (*p* = 0.97), indicating that magnetic field strength was not a major source of heterogeneity in the meta-analysis. Further confirmation through studies with larger sample sizes is warranted.

The ΔT1 values obtained with ShMOLLI were significantly higher than those acquired using the conventional MOLLI protocol (*p* = 0.01), suggesting that the pulse sequence may influence ΔT1 variations. Variants of MOLLI and ShMOLLI could show varying heart rate sensitivity, which could lead to variations in ΔT1 results during stress T1 mapping protocols (16, 17). MOLLI needs 17 heartbeats to collect data, and is limited by heart rate sensitivity (18). The ShMOLLI technique enables substantially reduced breath-holding durations, requiring merely 9 cardiac cycles for complete data acquisition (19). This improvement in efficiency arises from an integrated conditional reconstruction algorithm that effectively eliminates heart rate dependency during imaging (20). Burrage et al. (16) reported that the ShMOLLI sequence showed a stronger correlation with increased MBF under Regadenoson stress, contributed to enhanced T1 reactivity. Current guidelines recommend using imaging sequences with shorter breath-hold requirements, as they are less susceptible to cardiac motion artifacts compared to those requiring prolonged breath-holding (17). Although the increased heterogeneity in ΔT1 measurements could theoretically stem from distinct technical characteristics of the MOLLI and ShMOLLI protocols, definitive conclusions await rigorous methodological comparison through controlled experimental validation.

In the subgroup analysis of pharmacologic stress agents in studies utilizing ShMOLLI, no statistically significant differences were observed between the regadenoson and adenosine groups (*p* = 0.22), suggesting that the type of vasodilator agent is unlikely to be a major source of heterogeneity. Current evidence remains inconclusive as to whether different classes of loading medications exert differential effects on T1 reactivity. *In vitro* experimental data demonstrate that regadenoson exhibits superior vasodilatory efficacy compared to adenosine, with enhanced selectivity for coronary circulation in animal models (21, 22). Comparative studies show that regadenoson induces more pronounced hemodynamic stress on MBF and MPR than conventional agents such as adenosine and dipyridamole. However, mechanistic analyses reveal these enhanced perfusion parameters are mediated not through augmented vasodilatory potency, but rather by regadenoson's unique capacity to elicit more robust heart rate elevation responses (23–25). The administration of adenosine poses challenges for MOLLI-based T1 mapping techniques due to its significant chronotropic effect. This agent typically increases heart rate by 30–40 beats per minute (bpm), a physiological response that can introduce confounding artifacts in T1 relaxation times measured using the MOLLI sequence. The heart rate-dependent nature of the MOLLI acquisition makes it particularly susceptible to pharmacologically induced tachycardia, potentially compromising measurement accuracy by altering myocardial tissue characterization parameters (17). Overall, the association between stress T1 mapping and heart rate is an intriguing topic that warrants further investigation through comparisons of various T1 mapping techniques and pharmacological stress agents.

The study population consisted predominantly of males (56.19%), suggesting a possible gender bias. To date, no confirmed effect of age or gender on ΔT1 has been established. The influence of gender on myocardial perfusion remains controversial. In a study of healthy volunteers (26), adenosine stress CMR revealed higher myocardial perfusion and MBV in females compared to males, indicating that sex is an independent determinant of cardiac perfusion. However, a study by Range et al. (27) observed no gender-related differences in perfusion under adenosine stress. Although age was identified as a source of heterogeneity in pooled ΔT1 values across experimental groups in the present study, further experimental-level investigations are needed to clarify its impact on ΔT1.

Currently, stress T1 mapping is not yet standardized. Pending the release of formal guidelines, we recommend referring to existing recommendations on T1 mapping (28). Reference ranges for ΔT1 should be established using institution-specific control groups. Furthermore, since this meta-analysis demonstrated a significant effect of age on ΔT1 values, we advise including age as a stratification factor in participant selection for future studies. As sequence parameters become increasingly standardized across sites and vendors, broader normal ranges—or at least ranges specific to scanner vendors—could be developed.

Although experimental studies have demonstrated the strong potential of ΔT1 in detecting myocardial ischemia, clinical studies have reported inconsistent findings, leaving its true diagnostic value unclear. This discrepancy may be attributed to the limited number of available studies, their generally small sample sizes (*n* < 50), and the complexity of comorbidities within study populations (29). These factors substantially challenge the reliability of statistical conclusions. Therefore, the objective of the present study is to perform a meta-analysis synthesizing existing research on ΔT1 in healthy individuals. The integration of multi-source data serves to mitigate the constraints of small sample sizes and reduce confounding by comorbidities, thereby facilitating a more precise assessment of heterogeneity sources affecting ΔT1. We expect that this work will help clarify the clinical relevance of T1 reactivity and contribute to the advancement of its application.

Our study has several limitations, some of which are common to meta-analyses and others specific to our work. It is extremely important to know that *I*^2^ does not suggest whether or not the observed heterogeneity is clinically relevant; it only confirms the existence of the heterogeneity (30–32). At this stage, it remains unclear whether the variations across trials reflect clinically meaningful differences. Additionally, a key caution is that the overall summary results may be marked by substantial heterogeneity. The pooled means are limited in their suitability as normal reference values, as they were not derived from patient-level data. Similarly, the confidence intervals should not be interpreted as the upper and lower bounds of a reference range (33). Although meta-regression techniques were employed, the results should be interpreted with caution and viewed as hypothesis-generating, given the lack of access to original patient-level data.

The meta-analysis included a limited number of studies, which makes the findings susceptible to potential biases (34). Most of the studies had small sample sizes, often comprising fewer than 50 participants. Although subgroup analyses and meta-regression were performed, these factors likely contributed to the observed heterogeneity in the results. Since the studies were conducted on healthy individuals, the majority did not provide comprehensive baseline characteristics or apply uniform inclusion criteria, leading to lower quality scores for some articles. Because of nonuniform reporting, we could not specifically investigate variables such as heart rate, which may be of importance because some pulse sequences have possible heart rate dependence. Since the equipment utilized in the study and reviewed here was from the same supplier, it was not possible to compare the differences in ΔT1 between the vendors. Previous research (35) has indicated that a flip angle of 50° is associated with lower native T1 values compared to a flip angle of 35°. However, since the flip angle was fixed at 35° in the studies analyzed here, its influence on ΔT1 could not be evaluated. Collectively, these factors may explain the heterogeneity observed in the pooled ΔT1 estimates.

Through a comprehensive analysis of the current stress T1-mapping literature, we calculated the mean and confidence interval of ΔT1 using data extracted from 10 articles. The pooled mean of ΔT1 in healthy adults exhibited substantial heterogeneity, which remained significant even after adjusting for multiple factors. Part of this heterogeneity was attributed to differences in pulse sequence and age. Finally, we emphasize the importance of standardizing cardiac MRI protocols and investigating factors influencing T1 reactivity.

## Data Availability

The original contributions presented in the study are included in the article/Supplementary Material, further inquiries can be directed to the corresponding authors.
