# Supplementary figures and images for: CMR T1 reactivity in healthy adult hearts: a systematic review and meta-analysis
Source: Front Cardiovasc Med. 2025 Nov 13;12:1627908. doi: 10.3389/fcvm.2025.1627908 (PMC12657357; doi:10.3389/fcvm.2025.1627908)

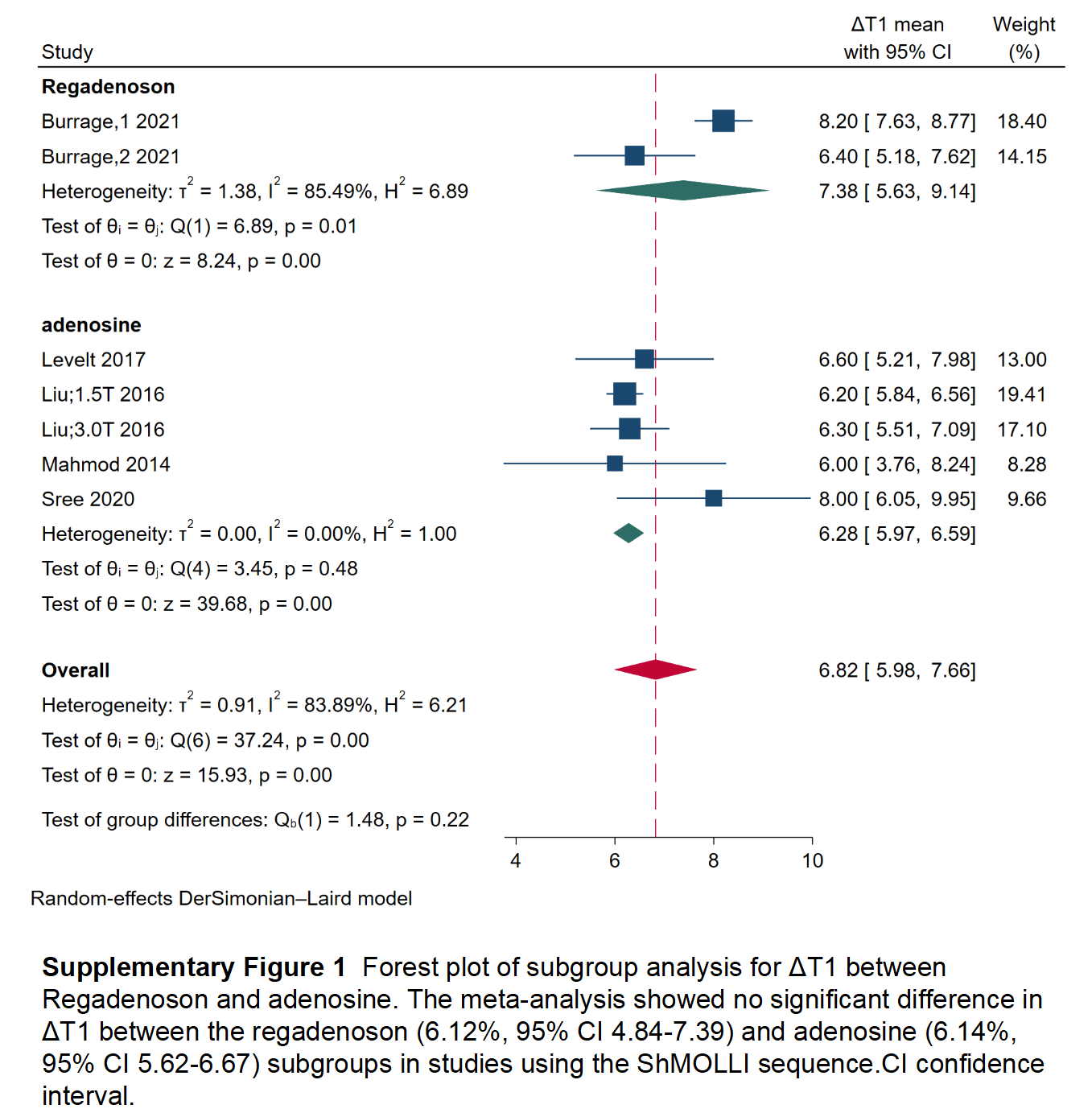

Supplement: Supplementary file 1 [file Image1.tif]

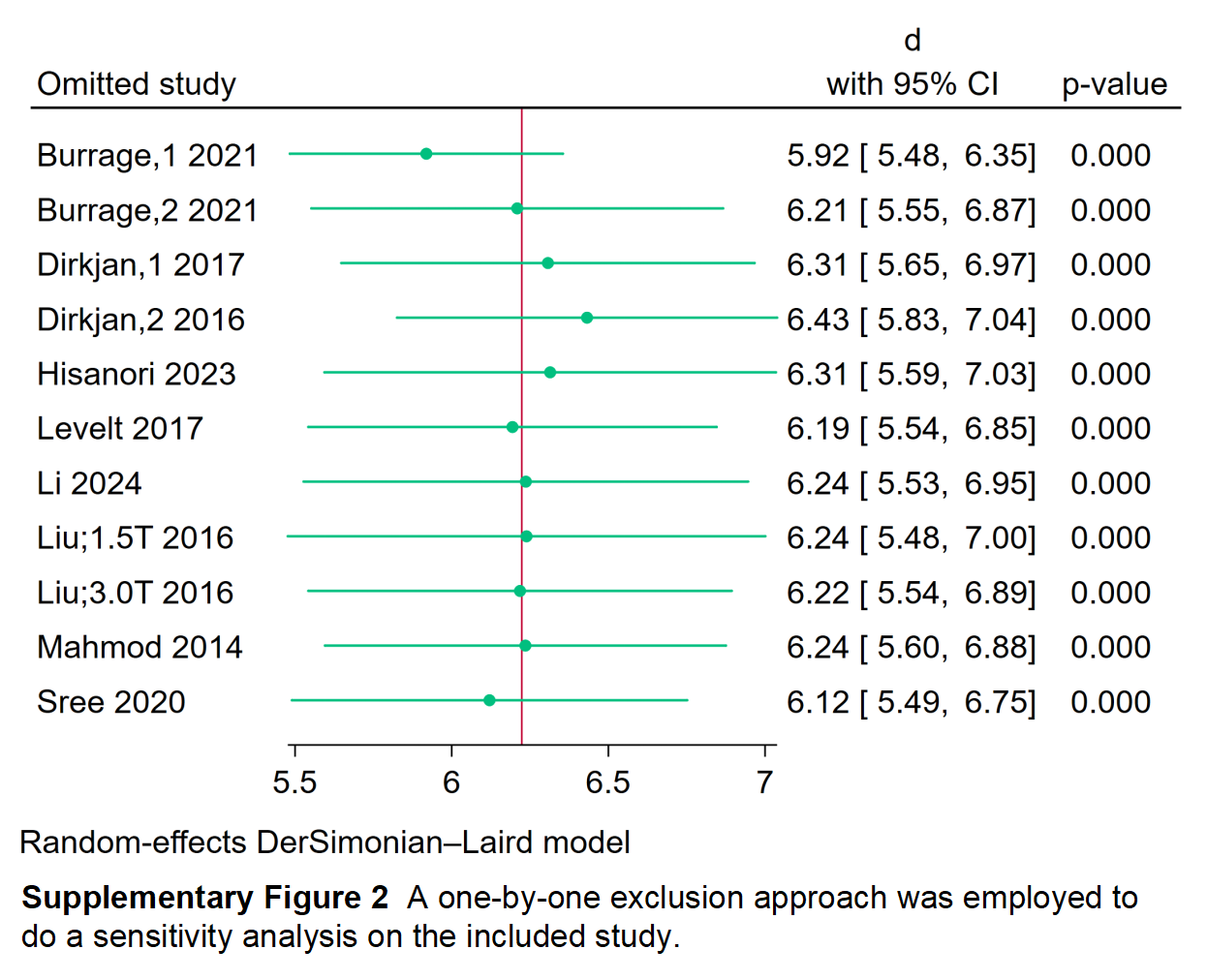

Supplement: Supplementary file 2 [file Image2.tif]
